# Identifying understudied correlations between autism & phenotypic attributes in a large family dataset

**DOI:** 10.1186/s40359-025-02739-4

**Published:** 2025-05-26

**Authors:** Maya McNealis, John Kent, Kelley Paskov, Kaitlyn Dunlap, Jordan Lane, Brittani Phillips, Melissa Armstrong-Brine, Shanna Kralovic, Anastasia Dimitropoulos, Leonard Abbeduto, Dennis P. Wall

**Affiliations:** 1 Departments of Pediatrics (Clinical Informatics), Biomedical Data Science, and Psychiatry and Behavioral Sciences, Stanford University, Palo Alto, CA USA; 2 Department of Psychological Sciences, Case Western Reserve University, Cleveland, OH USA; 3 MIND Institute and Department of Psychiatry and Behavioral Sciences, University of California Davis Health, Sacramento, CA USA; 4 Division of Child and Adolescent Psychiatry and Psychology, MetroHealth Medical Center, Cleveland, OH USA; 5 Division of Developmental and Behavioral Pediatrics and Psychology, Department of Pediatrics, UH-Rainbow Babies and Children’s Hospital, Cleveland, OH USA

**Keywords:** Autism, ASD, GI, Sleep, Seizures, Infections, Broad autism phenotype, Family study

## Abstract

**Background:**

Autism Spectrum Disorder (ASD), a neurodevelopmental condition marked by restricted, repetitive behaviors and social communication difficulties, is one of the fastest-growing pediatric behavioral health concerns in the United States. Long-term outcomes significantly improve with early intervention, but diagnosis and treatment are complicated by the large range of phenotypic presentations that can be moderated by identity factors like gender and culture. Many physical and behavioral characteristics associated with the autism phenotype are not included in the screening and diagnostic instruments used in research.

**Methods:**

We have built a multi-site registry of diverse families with children with autism to collect longitudinal data on their physical and behavioral attributes to study the heterogeneous autism phenotype. Our KidsFirst registry contains 6,951 participants (hereafter “children”) from 4,120 families, 1,865 of which have more than one child. In addition to collecting standard clinical instruments such as the Social Communication Questionnaire (SCQ), we have collected information on the phenotypic attributes of hearing issues, noise sensitivity, vision challenges, irregular sleep, impaired motor skills, metabolic disorders, gastrointestinal (GI) problems, infections, seizures, and premature birth for both ASD and non-ASD children. After validating parent-reported diagnoses against SCQ scores, we analyzed the association of each attribute with the ASD diagnosis and the other attributes using a logistic regression model and permutation tests.

**Results:**

Noise sensitivity, impaired motor skills, irregular sleep, GI problems, infections, and seizures attributes were significantly associated with autism diagnosis. These attributes also share correlation structures amongst themselves, suggesting that groupings of attributes may help to define subtypes of autism.

**Limitations:**

The attributes analyzed in this study are not a comprehensive list of suspected traits of autism. Parent-reported diagnoses may not always be accurate, although we validated diagnoses. Despite accounting for family structure in our experiments, the relationships between attributes and diagnosis are likely stronger in the general population because our “control” sample is comprised of biological siblings who may still possess subclinical autistic traits, given the heritability of autism.

**Conclusions:**

A more expansive conceptualization of the autism phenotype is likely to be useful to both researchers and families for identifying a more targeted approach to intervention.

**Supplementary Information:**

The online version contains supplementary material available at 10.1186/s40359-025-02739-4.

## Background

Autism spectrum disorder (ASD), hereafter referred to as autism and ASD, is defined by neuropsychological and behavioral symptoms, including the diagnostic features of social communication deficits and restricted and repetitive behaviors [1]. Autistic individuals experience comorbid challenges at a higher rate than the general population [2], including attributes such as verbal skills that extend beyond social communication [3, 4], hearing issues [5], noise sensitivity [6], vision challenges [7], irregular sleep patterns [8, 9, 10], impaired motor skills [11], metabolic disorders [12, 13], gastrointestinal problems [14, 15, 16], recurring infections [17], seizures [18, 19] and premature birth [20, 21]. We refer to these attributes as such to distinguish them from the formal criteria of other DSM-5 diagnoses which can be clearly differentiated from autism with standardized assessment.

## Measurement gaps in gold standard instruments

Despite the increased prevalence of these challenges in the autistic population, major autism diagnostic instruments such as the Autism Diagnostic Interview—Revised (ADI-R) [22], Autism Diagnostic Observation Schedule, Second Edition (ADOS-2) [23], and Social Communication Questionnaire (SCQ) [24] do not fully capture these attributes. Furthermore, the specific attributes parents may notice and describe when identifying their child’s autism phenotype vary with demographics like child age, sex/gender [25, 26, 27] and culture [28, 29, 30]. This makes it difficult to understand whether these attributes are merely elevated in autism, or whether they are an integral part of a more comprehensive and holistic conceptualization of autism that we refer to as an expansive autism phenotype, indicating a need for more diverse and large dataset analyses.

### Autism attributes

Many of these attributes are known to have relationships with features of the core autism phenotype and with each other. For example, motor skills have been shown to be associated with social skills [31] and expressive language [32] in autism. Both sleep disturbances and gastrointestinal problems have been shown to correlate with severity of autism symptoms [8, 16], and common genetic variants link the gut-brain axis to both prematurity and autism [33]. Auditory processing differences associated with autism are known to contribute to noise sensitivity, which can manifest in many different ways in autistic individuals, including feelings of overstimulation and difficulty with social communication in environments with ambient noise, as well as enhanced pitch detection and musical talent, exemplifying the heterogeneity and complexity of the autism phenotype [5]. Additionally, noise sensitivity is often linked to sensory processing disorder in individuals with ASD, where heightened sensitivity to auditory stimuli can lead to significant distress and functional impairment [34, 35, 36]. Furthermore, the interplay between noise sensitivity, overstimulation, and hyperacusis underscores the need for targeted interventions, as these factors collectively contribute to the challenges faced by individuals on the autism spectrum in their daily lives [37, 38].These findings suggest that autism-associated attributes have a correlation structure with the features of the core autism phenotype and with each other that may be helpful for identifying autism subtypes and individualizing treatment.

### KidsFirst dataset

We developed a rich phenotypic dataset capturing both the core features of autism, as well as a variety of attributes known to be associated with autism (hearing issues, noise sensitivity, vision challenges, irregular sleep patterns, impaired motor skills, metabolic disorders, gastrointestinal problems, recurring infections, seizures, and premature birth) through caregiver report. Our dataset is comprised of crowdsourced caregiver-reported information on a community sample of 6,951 children from 4,120 families who enrolled online into the KidsFirst registry. The goals of the present study were to (1) assess the feasibility of this type of crowd-sourced parent-report family study design for establishing a diverse and representative dataset (2) determine whether these attributes are significantly associated with autism in a large cohort and (3) identify the relationships between these attributes and autism diagnostic features to support the argument for expanding the autism phenotype and addressing these gaps in existing diagnostic tools.

## Methods

### Data collection

To develop a rich, diverse phenotypic dataset that could help interpret the heterogeneity of autism, The Hartwell Foundation created the KidsFirst initiative to recruit and crowdsource autistic families across the United States, with sites at Stanford University, University of California, Davis Health MIND Institute, and Case Western Reserve University and affiliated hospitals. Data were collected from May, 2017, to January, 2023.

The UC Davis Health MIND Institute used digital recruitment strategies, including email notices to participants in their online Research Participant Registry (RPR), national paid social media campaigns, and a KidsFirst description and referral link on the UC Davis Health MIND Institute website. Case Western Reserve University recruited participants in the Cleveland, OH area through University Hospitals and MetroHealth clinics, and conducted broader community recruitment across Northeast Ohio through in-person and online events. In-clinic participants were recruited in person prior to their scheduled appointments by either a developmental pediatrician, or a provider who specialized in neurodevelopmental conditions. Phone calls, emails, social media (Facebook), and texting were also utilized as recruitment methods for the Cleveland sites. Stanford University recruited participants via national paid social media campaigns, email campaigns to research lists maintained by Stanford that included families from across the U.S., and referrals from Stanford Autism Center (see summary of recruitment below).

#### UC Davis Health MIND Institute

- Email marketing to existing Research Registry.
- National social media campaigns.
- UC Davis Health MIND Institute Website.

#### Case Western Reserve University

- In-person recruitment at University Hospitals and MetroHealth Clinics.
- In-person recruitment at community events.
- Phone and Text Recruitment.
- Email via existing research registry.
- Social Media (Facebook) Posts.

#### Stanford University

- National social media campaigns.
- Email marketing to existing Research Registry.
- In-clinic referrals from Stanford Autism Center.

The KidsFirst registry contains detailed, demographic information about participating families, and survey responses collected from them. It is interfaced via a secure website (from any computer or mobile device browser), kidsfirst.stanford.edu, where participants could create an account, complete surveys, view their results in comparison to the registry population, and access or print their surveys at any time.

**Fig. 1:**
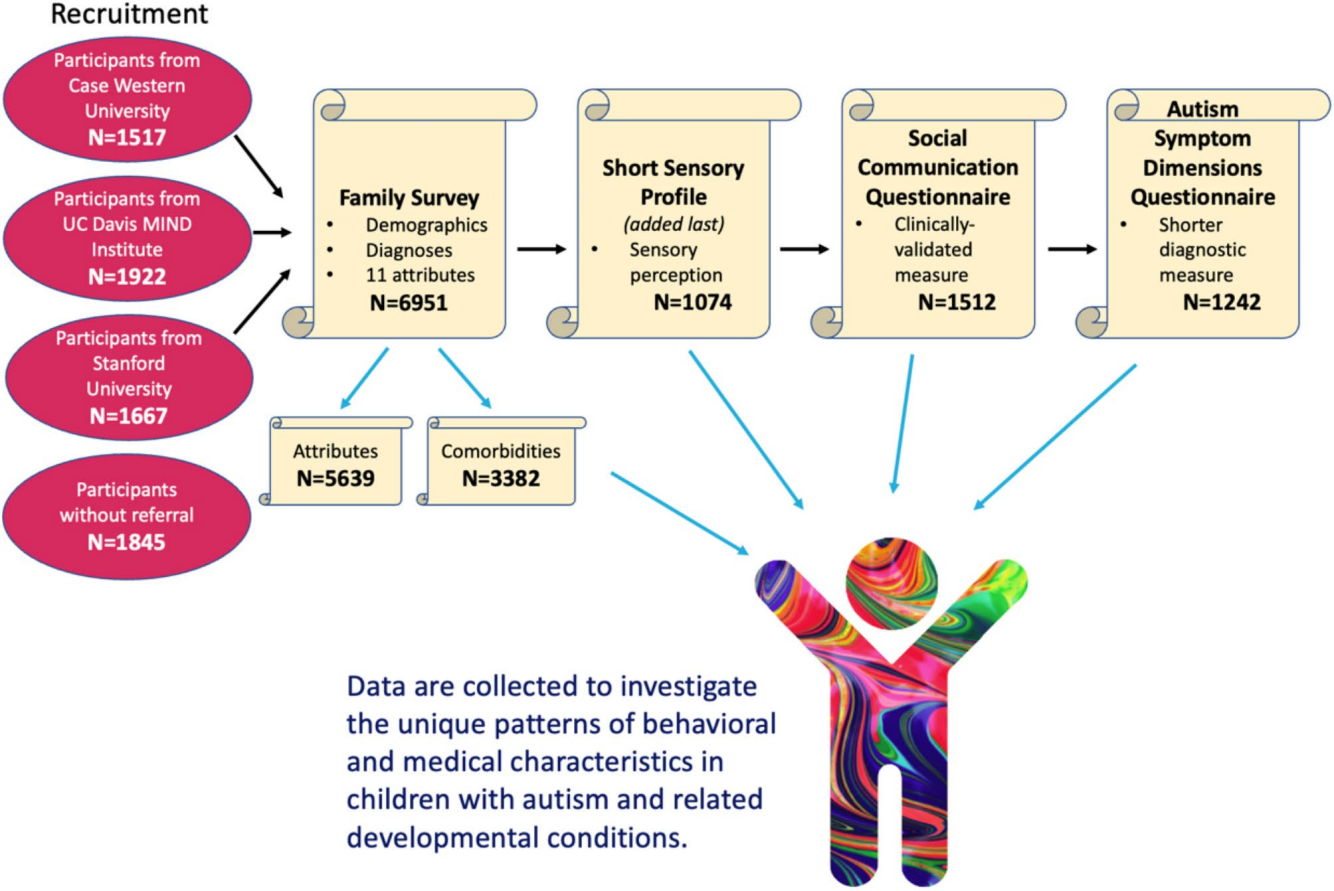
Study flow diagram depicting process of recruitment and data collection

The data collection process is shown in Fig. 1. After recruitment, families were asked to complete an intake survey that collected demographic information, parent-reported diagnoses, family structure, medical history, comorbidities, and details on 11 phenotypic attributes of interest chosen via clinical consensus, which we refer to as premature birth, verbal skills (verbal), irregular sleep (sleep), hearing issues (hearing), vision challenges (vision), noise sensitivity (noise), impaired motor skills (motor), metabolic disorders (metabolic), gastrointestinal problems (GI), recurrent infections (infections), and seizures, for each of their children. The questions about attributes asked of each family along with possible responses are shown in Table 1. This created a related record in the database for every child with and without autism in a family, and subsequent survey data were collected for every child. Storing survey data on all siblings in this manner, including their relatedness (full, half, adopted, etc.), allows us to leverage knowledge of family structure in validation and comparative analysis.

After the intake survey, families were invited to sequentially complete 4 additional surveys: the Social Communication Questionnaire, the Autism Symptom Dimensions Questionnaire [39], the Short Sensory Profile [40, 41], and (optionally) parent-measured head circumference supported by video instructions [42]. Additionally, they were invited to securely submit any clinical documents they wished to share as images, PDFs, or Microsoft Word documents. Parents received a $20 Amazon gift card for completing all surveys and could revisit the website to continue their progress or view their results at any time. The surveys were presented sequentially, and all form fields were required for each survey to be submitted, but because users were allowed to leave and return to the website on their own time to complete them, each survey had a smaller sample of completed responses than the ones prior. The Short Sensory Profile was added to the study design last, and so despite being the second survey in the sequence, it has the lowest number of responses (1,074). Because of this variation, the subsample sizes have been restated for each portion of our analysis.

While we actively recruited autistic families both through outreach at our partnered clinics and through online advertising, we had no exclusion criteria related to autism, and in 2022 we also began recruiting families from our partner clinics that did not have autism, but had been diagnosed with specific developmental conditions that overlap with autism in terms of comorbid phenotypic attributes, including attention deficit hyperactivity disorder (ADD/ADHD), speech and language disorders, anxiety disorders, and intellectual disability / global developmental delay, to enable us to compare families with ASD to a psychiatric control group. The survey was revised to include a question asking parents to indicate all conditions their child(ren) were diagnosed with from this list: speech/language disorder, attention deficit hyperactivity disorder (ADD/ADHD), anxiety, depression, intellectual disability/global developmental delay, motor/developmental coordination disorder, oppositional defiant/conduct/disruptive behavior disorder, feeding or eating disorder, specific learning disorder—written expression, specific learning disorder—reading, specific learning disorder—math, obsessive compulsive disorder (OCD), tic disorder/tourette’s, elimination disorder, and bipolar disorder/mania. All study materials were initially provided in English only, but partway through recruitment Spanish translations were made available for any interested families to assist in recruiting Spanish-speaking families.

**Table 1:**
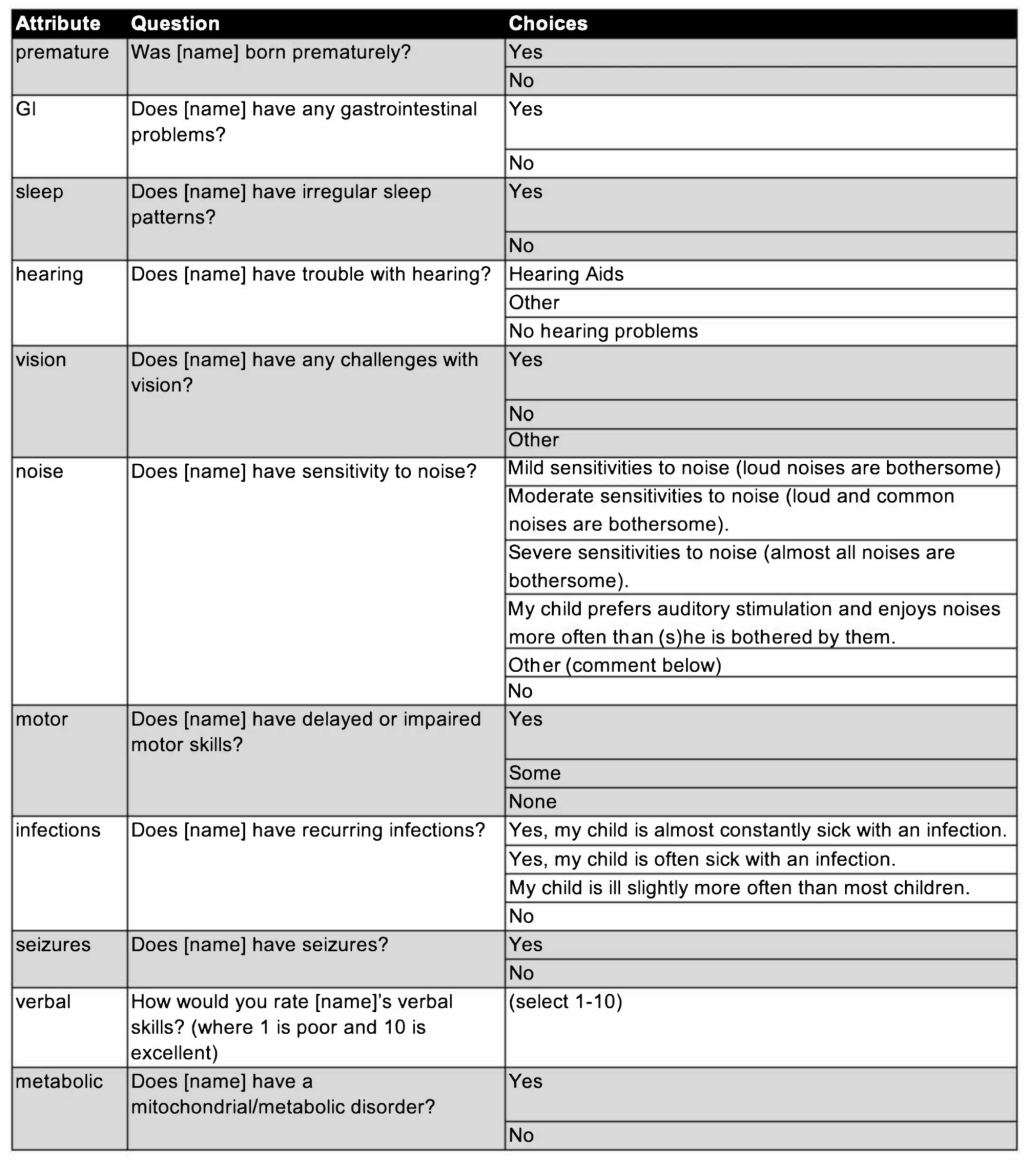
Questions and answer choices for the 11 attribute questions in the intake survey

### Statistical methods

#### Validation

Before analyzing the phenotypic data, we first validated parent-reported diagnoses using scores on the Social Communication Questionnaire, a clinically validated autism screening assessment (Supplementary Fig. 1). We compared the 1,512 SCQ scores of the various diagnostic groups (as reported by caregivers) using a Mann-Whitney U test due to the non-normal distribution of SCQ scores between the autistic, neurotypical (NT), and psychiatric control groups. Expert consensus and informal stakeholder interviews were used to validate the interpretability of survey questions. Additionally, we compared responses about prematurity with responses to weeks of gestation, another quantitative measure of the same trait, at every step of our analysis to ensure participants responded reliably.

#### Confounder analysis with logistic regression

Given that the median male-to-female ASD prevalence ratio is 4:1 [43], and several of our attributes of interest may coincide with developmental phases that correspond to age, we suspected that the sex and age variables could potentially confound our association results. To address this, we used a logistic regression model including age and sex as confounders because it enabled rigorous adjustment for crucial variables like sex and age, providing clearer insights into the true associations between attributes of interest and ASD prevalence. This test is preferable to alternatives such as simple univariate analyses that would fail to account for these confounders and potentially lead to biased estimates or more complex models that may not effectively isolate the specific effects of the variables of interest. We binarized the responses to the 11 attributes, counting them as “present” or “not present”, as follows: for verbal skills, any score less than or equal to 7 was counted as “present” as 7 is a common threshold on 10-point scales [44, 45, 46] and we observed high correlation between scores 0–7 and SCQ scores less than 15; for noise sensitivity, we counted all responses other than “no” as indicating a presence of this feature because we changed this question part way through to add additional options that addressed sensitivity as well as sensory seeking and our statistician determined it was best to assess this feature at a lower granularity (present or not present) in this instance of analysis; for all other attributes, any response other than “no” was considered “present”. For each pair of attributes, we used statsmodels to predict one attribute using the other attribute, age, sex, and an offset term. We then reported the coefficients, p-values, and confidence intervals calculated by statsmodels. Bonferroni was used for multiple test correction due to its stringent control of type-I error.

#### Permutation test

Our registry contains siblings, and it is possible that the attributes included in this analysis may have genetic components unrelated to autism. Therefore, to confirm the associations between each attribute and the autism diagnosis even under the family structure of our cohort, we ran a permutation test. Permutation tests are better suited for this analysis due to their ability to handle sibling dependencies, distributional assumptions, and outliers, making them an ideal choice for studying associations in genetic or familial contexts. We permuted sibling diagnoses within each family 12,000 times (above the number needed for establishing a null distribution) and calculated the number of times the chi-square test statistic of the permuted data was as or more extreme than the reported data to produce a *p*-value. We ran this permutation test for each attribute to establish the statistical association between the attribute and autism.

## Results

### Overview

The study analyzed data from nearly 7,000 children, identifying significant associations between ASD and various attributes, such as noise sensitivity, impaired motor skills, and irregular sleep. Specifically, children with ASD were more likely to experience heightened sensitivity to noise and difficulties with motor skills compared to those without ASD. Importantly, factors like gastrointestinal problems, infections, and seizures also showed strong links to autism. Key findings indicate a robust connection between noise sensitivity and impaired motor skills with ASD. Moreover, attributes such as irregular sleep, and gastrointestinal problems were also identified as common among children diagnosed with ASD. These relationships were maintained even when family relations (siblings in the dataset), age, and sex, were considered. The demographic analysis revealed that 30.8% of the children in the study were female, although only 23.4% of the children with autism diagnoses were female. Additionally, the median age of the children in the study was 11 years, and the sample was diverse, with 33.6% identifying as non-white.

### Demographics

**Fig. 2:**
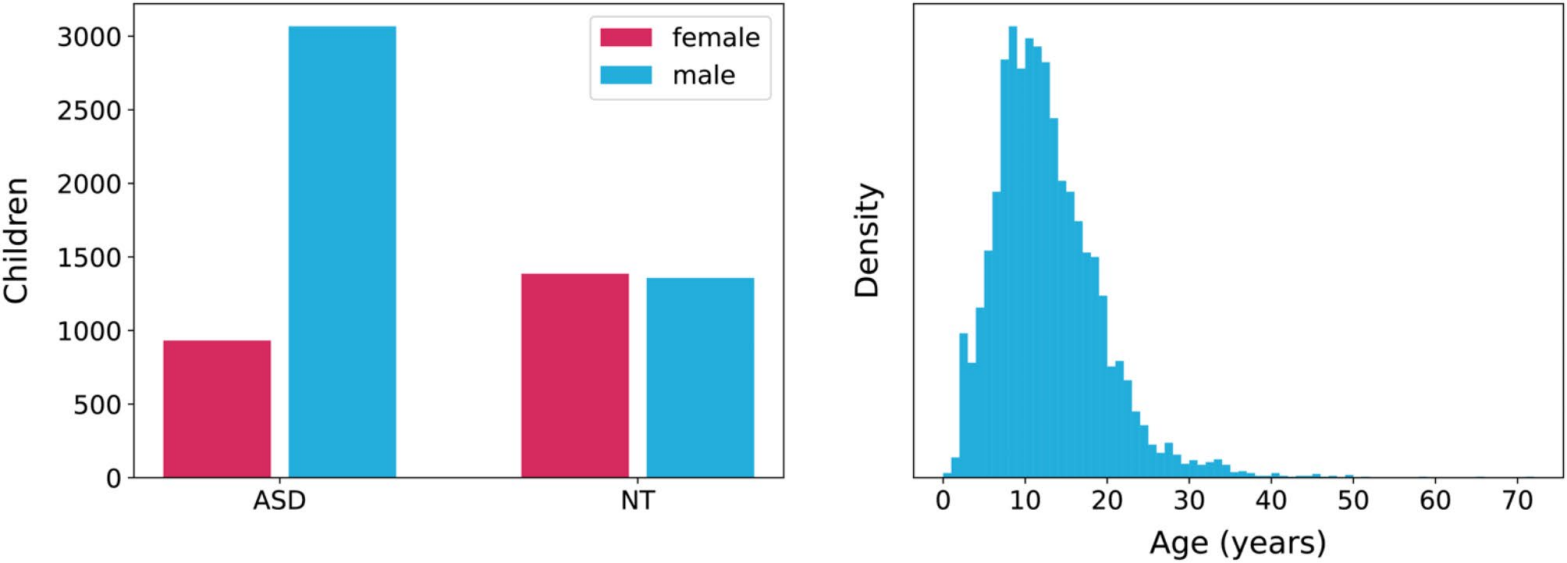
Sex and age distribution for children in our registry. 76.6% of the children with ASD in our dataset are male, corresponding to the male: female ratio of ASD. The median age of children in our registry is 11 years

We collected data on 6,951 children, 5,639 of whom completed the attribute measures analyzed in this paper. Of these children, 4,002 have autism diagnoses and are labeled “ASD group” in our analysis. 774 have no diagnoses and 872 have other, non-autism conditions; together these samples are referred to as the “non-ASD group”. 30.8% of children in the dataset are female, and 23.4% of children with an autism diagnosis in the sample are female as shown in Fig. 2. The median age of children in our sample is 11 years as shown in Fig. 2. The sample includes 1,194 adult children age 18 or older living with a parent, guardian or caretaker. The sample is diverse, with 33.6% identifying as non-white (See Table 2 below for full demographic breakdown) and 21.41% of our families earning less than $35,000 in pre-tax income.

**Table 2:**
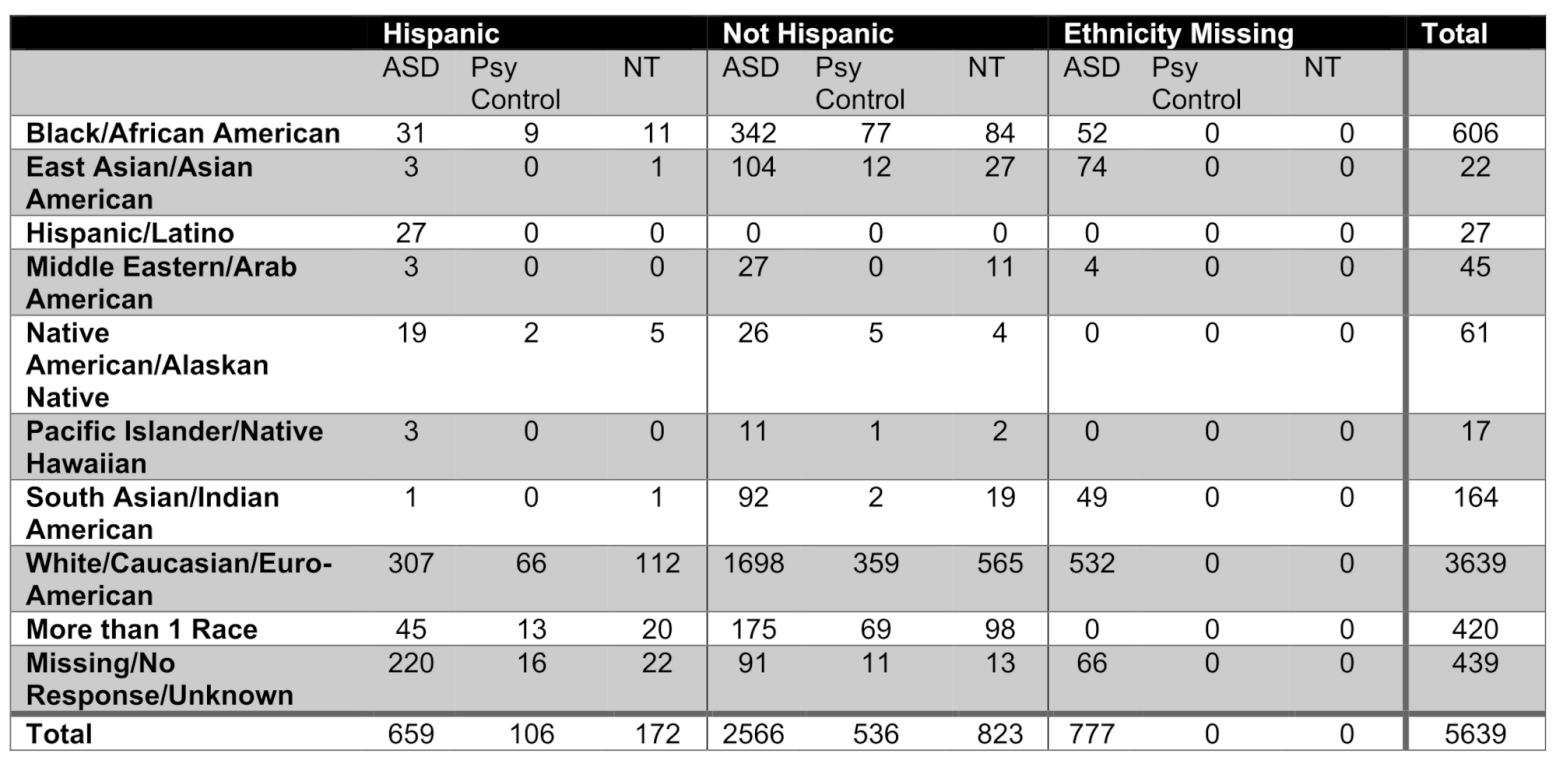
Full demographic breakdown for the sample analyzed by race and ethnicity and diagnosis groups

### Comorbidities

In addition to autism diagnosis, parents were asked to report on the presence of 15 additional diagnoses or conditions for each of their children as shown in Table 3. This comorbidities question was added to the survey after some data had already been collected, so the subsample of surveys containing comorbidities data is 3,382. Among the ASD group, the most common comorbid diagnoses are speech/language disorder (33.04%), attention deficit hyperactivity disorder (30.79%) and anxiety (23.69%). The most common diagnoses among the non-ASD group are attention deficit hyperactivity disorder (26.41%), anxiety (20.05%) and speech/language disorder (17.69%).

**Table 3:**
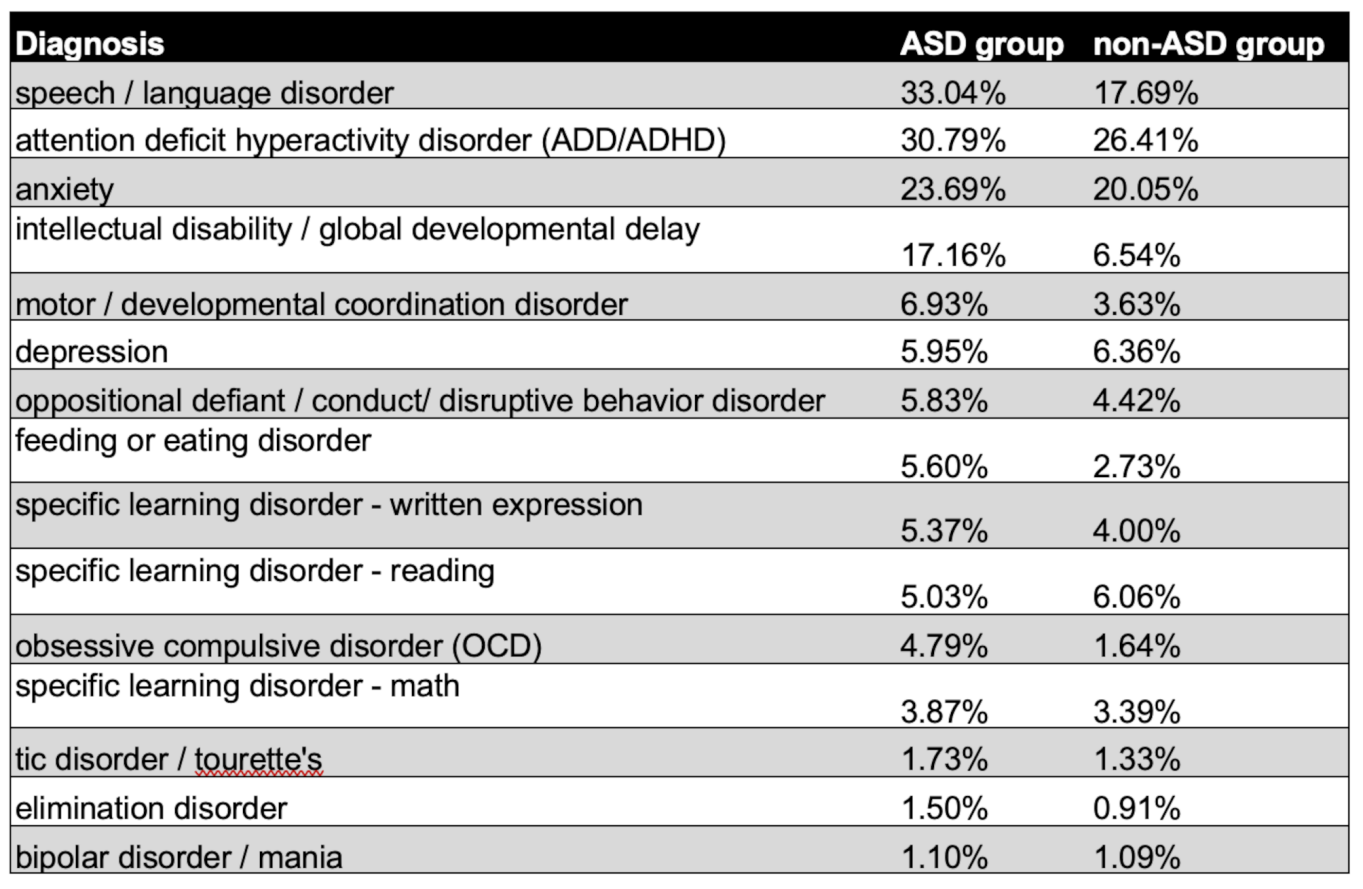
The prevalence of comorbidities in the ASD and non-ASD groups. Intellectual disability/global developmental delay is underestimated due to the fact that this question was added to the study design partway through data collection

### Summary of attributes

**Fig. 3:**
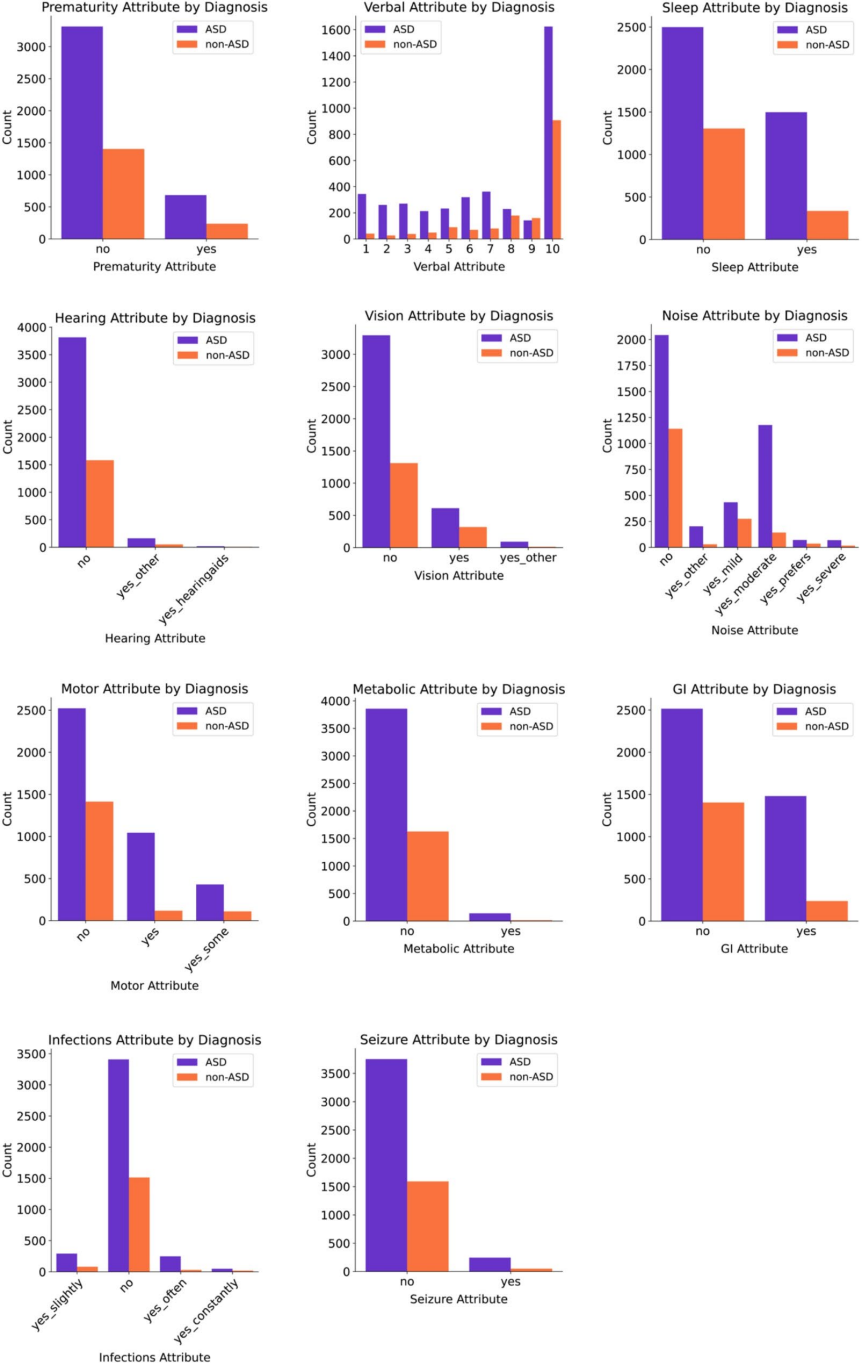
Attribute distributions are shown, split by ASD diagnosis. Counts, rather than proportions, are shown to better communicate sample sizes. Prematurity, sleep, metabolic, GI and seizure responses are binary. Vision, hearing, motor, noise sensitivity and infections responses are categorical. Verbal skills responses are discrete. There is representation across multiple answer choices for each attribute for both ASD and non-ASD diagnoses

Table 4 displays the prevalence of each of the 11 attributes in our sample. The most prevalent attributes in the ASD group are verbal skills (50.09%), noise sensitivity (48.89%) and impaired motor skills (36.88%). The most prevalent attributes in the non-ASD group are noise sensitivity (30.51%), verbal skills (24.06%) and vision challenges (20.16%). Figure 3 Displays the attribute response distributions split by autism diagnosis.

**Table 4:**
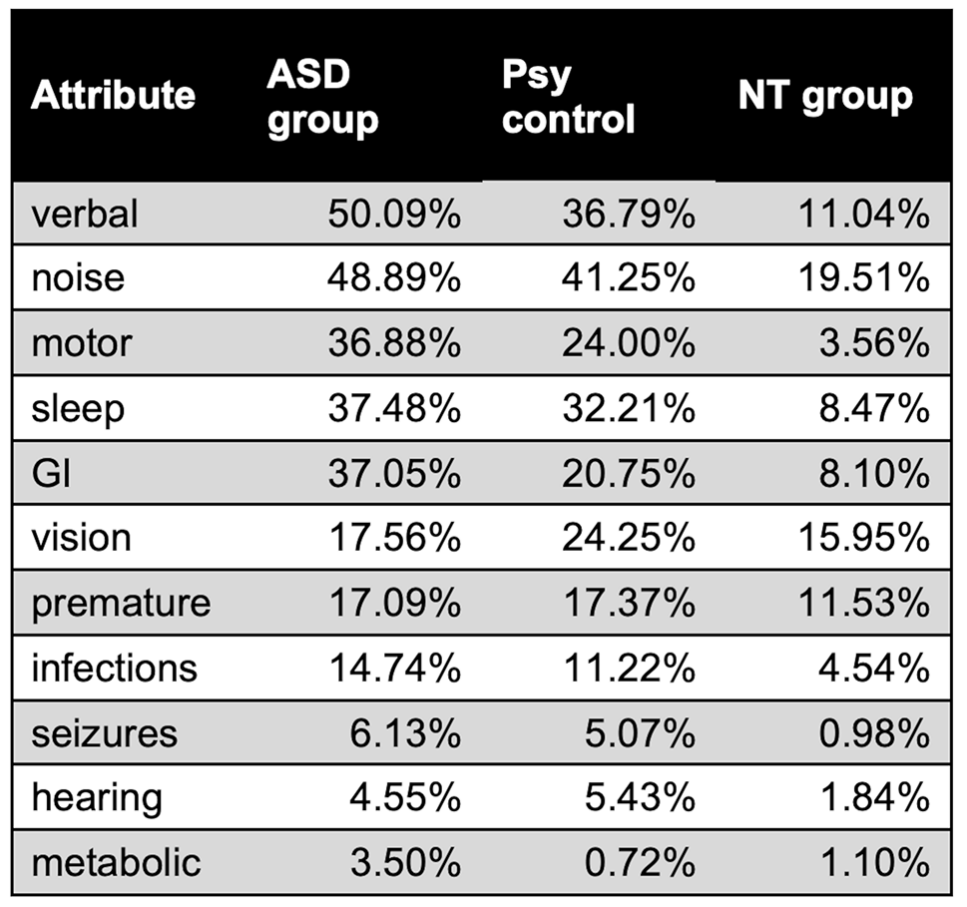
The prevalence of each attribute in the ASD sample, other psychiatric diagnoses sample and the NT sample

### Social Communication Questionnaire Validation

Given that parent-reported diagnosis is validated by SCQ data (Supplementary Fig. 1) and parent-report of autism diagnosis has been validated in previous work [47, 48, 49], we can reliably assess autism features with this dataset. As an additional validation, we confirmed that prematurity data correlated with weeks of gestation, another quantitative measure of the same trait, at every step of our analysis.

### Confounder analysis with logistic regression

**Fig. 4:**
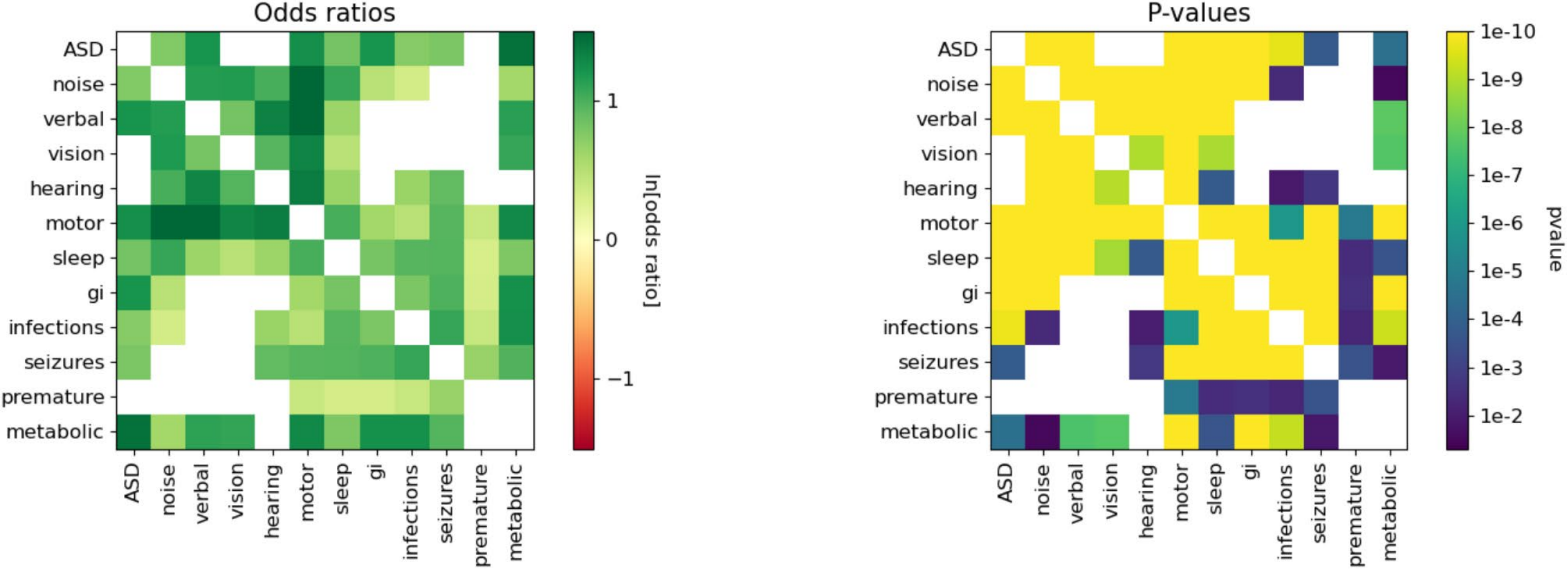
Odds of the attribute associations. The heatmap on the left displays the odds of the associations between attributes for the full sample, adjusted for age and sex. Green corresponds to a positive association, and red corresponds to a negative association. All associations were positive. The heatmap on the right displays the Bonferroni corrected significance level of each relationship. Yellow squares representing the highest confidence level. White squares in both plots indicate no significance in the associations

The attributes have significant correlations with both autism and each other as shown in Fig. 4, even after correcting for the presence of sex and age as confounders. Noise sensitivity, motor, sleep, GI, verbal skills, infections, seizures, and metabolic attributes are all significantly associated with the ASD diagnosis. Hearing issues and prematurity are not significantly associated with autism diagnosis in our data.

Furthermore, many pairs of attributes show significant association with each other as shown in Fig. 4. Classical ASD phenotypes such as noise sensitivity, motor, and verbal skills show many pairwise associations with each other as well as with vision and hearing. GI, infections, and seizures also show many pairwise associations with each other. Sleep and motor share significant associations across both groups.

**Fig. 5:**
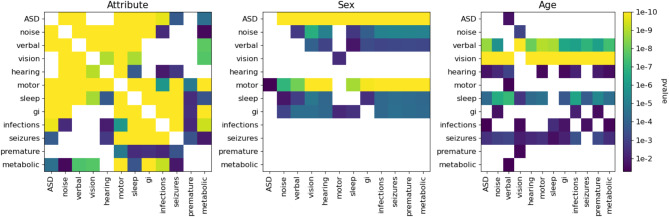
Predictive contribution of attributes and confounders. Together, these three matrices display the predictive ability of our features (phenotypic attributes and diagnosis), sex, and age on each attribute and diagnosis

Figure 5 shows the significance of sex and age in the logistic regression model. Sex is significantly associated with ASD diagnosis, as expected. Age is highly associated with the verbal skills and vision attributes.

### Permutation test

Permutation tests confirmed that many of the 11 attributes are indeed associated with autism diagnosis even after controlling for family structure (Table 5). The attribute-diagnosis associations remained for noise sensitivity, motor, sleep, GI, verbal skills, infections and seizures. The association between the metabolic attribute and autism diagnosis was not more extreme than the permuted statistic at the 95% confidence level.

**Table 5:**
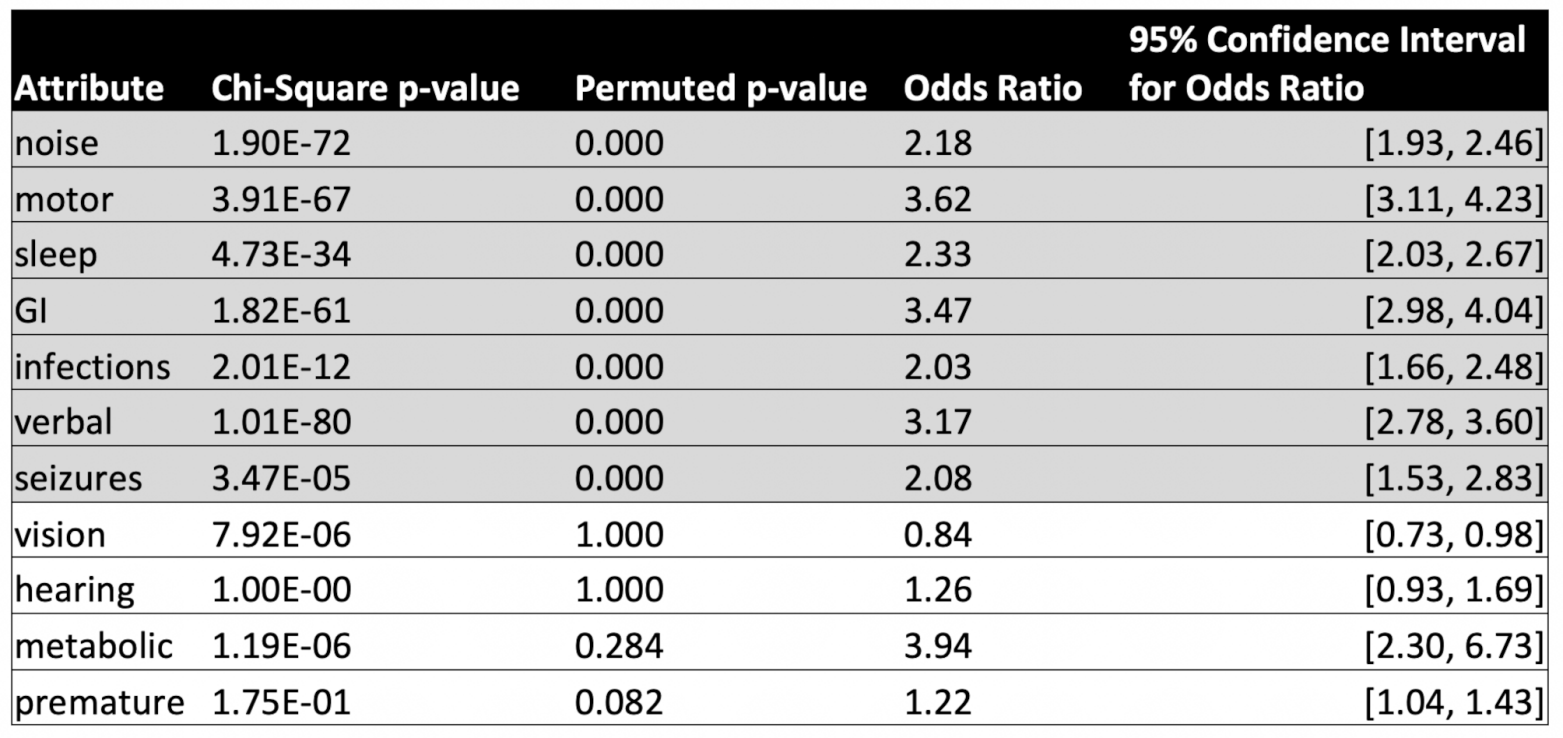
The odds of a child having each attribute if they also have ASD compared to if they do not have ASD are included, with 95% confidence intervals. Attributes which had ASD associations confirmed by the permutation test and odds ratio confidence intervals are highlighted.

## Discussion

The autism phenotype captured in the KidsFirst registry is broad and diverse with respect to sex, age, ethnicity and presence of comorbidities. Parent-reported diagnosis has been shown to agree with standardized measures for pediatric autism research [50], and SCQ validation confirmed significant differences between our autistic, NT, and psychiatric control samples. Our confounder analyses confirmed that the correlations we identified were consistent across sex and age.

As expected, classical features of the autism profile (noise sensitivity, impaired motor and verbal skills attributes) are significantly correlated with autism diagnosis, lending integrity to our analysis. Our work is in line with existing literature on the high prevalence rates of sleep dysfunction [51, 52, 53], GI problems [54, 55, 56, 57], seizure disorders, speech and language delays [58, 59, 60, 61, 62], and sensory processing difficulties [36, 63, 64], and motor coordination deficits [65, 66]. The associations observed here between sleep, GI, seizures and infection attributes and autism diagnosis suggest that further research into these attributes in autistic populations could expand the autism phenotype and potential treatment opportunities. Even the weak association between the metabolic attribute and autism, which likely reflects the small subsample of participants with metabolic disorders in our dataset, may support expanding the autism phenotype as the association could be stronger in larger samples [67, 68]. The absence of a relationship between prematurity and autism diagnosis may warrant a reexamination of the previously established relationship between premature birth and this neurodevelopmental condition [69]. Follow-up studies that leverage large electronic health record datasets or characterize autism dimensionally could better quantify the association between prematurity and autism diagnosis.

Given the inheritance patterns of autism, it is also possible that the sibling controls included in our family study are exhibiting subclinical traits of the broad autism phenotype [70, 71, 72] and thereby dampening the associations detected in our analysis, causing us to underestimate the association between prematurity and the autism phenotype.

Several of these attributes (verbal skills, noise sensitivity, impaired motor skills, irregular sleep, GI) produced a signal strong enough to suggest that we could deepen our understanding of autism through assessing noise sensitivity, motor challenges, irregular sleep, and GI problems in addition to the criteria listed in the DSM-5. An understanding of additional phenotypic traits of autism could help address communication gaps between affected individuals and their families or caregivers to produce more effective treatment approaches. Expanding the attributes assessed in the autism diagnostic process stands to benefit autistic individuals by allowing for better intervention selection, and a more data-driven approach to tracking progress over time. For example, our findings on noise sensitivity contribute to ASD subtyping work by highlighting how there may be distinct sensory profiles that can inform tailored intervention strategies, allowing for more personalized treatment approaches based on individual sensory sensitivities [37, 38, 63, 73]. Future work assessing each attribute’s onset could help identify timepoints at which prediction of autism and risk scores could be created, based on the presence or absence of associated attributes. Predictive and longitudinal modeling approaches would have the added benefit of identifying subtypes of the autism phenotype, based on various combinations of attributes.

These results are preliminary and should be considered accordingly. This initial examination of attribute associations may lead to follow up analysis of differences in children with various attribute combinations. Validation of these findings using a large-scale electronic health record analysis is an important future pursuit. Future work with a larger dataset may also uncover additional attributes that ought to be included to comprehensively characterize the autism phenotype. The granularity we captured in questions about verbal skills, hearing issues, impaired motor skills, noise sensitivity and infections attributes also provides a starting point to examine the relationship between the severity of these challenges and the autism phenotype. The potential to leverage both dimensional and categorical approaches to this dataset is a unique and compelling feature we hope to explore in future work. Our next analyses will initiate this effort by exploring the predictive capabilities of our attribute data to begin identifying possible groupings to expand the heterogenous autism phenotype.

### Limitations

This study has several limitations. First, we did not confirm autism diagnosis beyond parent-report and SCQ validation. We did not include instruments that validated comorbidity diagnoses, relying on parent-report for those data. This limitation could be resolved in future work by cross-validating diagnostic labels and attribute reporting using both clinician and parent-report to address variability in parent reporting. Baseline agreement levels could help ensure the sample does not contain any misreported data. Second, our attribute survey collects very high-level information on each of the 11 attributes. It would be good to assess these attributes in more detail and with more sensitivity in future work. Our future work will include anchor points to assist participants with accurate reporting, and noise sensitivity-related and vision-related diagnoses to enable better comparison of attributes and children’s formal diagnoses. Third, it is likely that parents define and report severity of their children’s attributes very differently. However, because we collected attribute data for families with multiple children, we are still able to capture relative differences between children within one family and can therefore still draw meaningful conclusions about the ASD versus non-ASD groups. Fourth, some of our attribute subsamples, like metabolic, are small enough that our association analyses may not have detected the true signal between the attribute and diagnoses. Future work could identify whether any true signal is found in the relationship between metabolic conditions and autism through working with a larger sample or specifically recruiting those with metabolic conditions in a dedicated study. Fifth, our selection of attributes is not exhaustive, and there are likely other traits relevant to the autism phenotype that should be assessed to fully define different autism phenotypes. Sixth, there were geographical constraints to our recruitment process. Although families across the US were eligible to participate, we specifically recruited families at 3 sites. While our sample is diverse with respect to child age, sex, race/ethnicity, and socioeconomic status, there is a possibility our conclusions are not representative of the broader population of families with autism. Continued efforts to make online participation accessible and as simple as possible will help ensure broader participation in future work.

## Conclusions

Defining a more expansive autism phenotype that includes attributes like sleep, GI, infections and seizures may aid in better understanding autism. Expanding the autism phenotype may also help direct autistic people and their caregivers towards treatments and resources that improve associated symptoms. Primary care physicians and others providing healthcare to autistic individuals may also benefit from knowing these associations such that they are able to tailor their evaluations and monitoring accordingly. Integrating sleep, GI, infections and seizure attributes into clinical frameworks of autism would help translate this research into clinical settings. Furthermore, illuminating phenotypic associations within the autism profile may be useful for characterizing subtypes of autism, leading to more personalized and effective neurodevelopmental diagnostic approaches in the future.

## Electronic supplementary material

Below is the link to the electronic supplementary material.

Supplementary Material 1

## Data Availability

The datasets generated and analyzed during the current study are available upon request at kidsfirst@stanford.edu. Competing interests. The authors have no competing interests to declare.

## Abbreviations

ASD: Autism Spectrum Disorder
NT: Neurotypical
ADHD: Attention Deficit Hyperactivity Disorder
SCQ: Social Communication Questionnaire
GI: Gastrointestinal
