# Supplementary material for: Identifying understudied correlations between autism & phenotypic attributes in a large family dataset
Source: BMC Psychol. 2025 May 26;13:561. doi: 10.1186/s40359-025-02739-4 (PMC12105231; doi:10.1186/s40359-025-02739-4)
Supplement: Supplementary file 1 — Supplementary Material 1 [file 40359_2025_2739_MOESM1_ESM.docx]

**Supplementary Materials:**

SCQ Validation


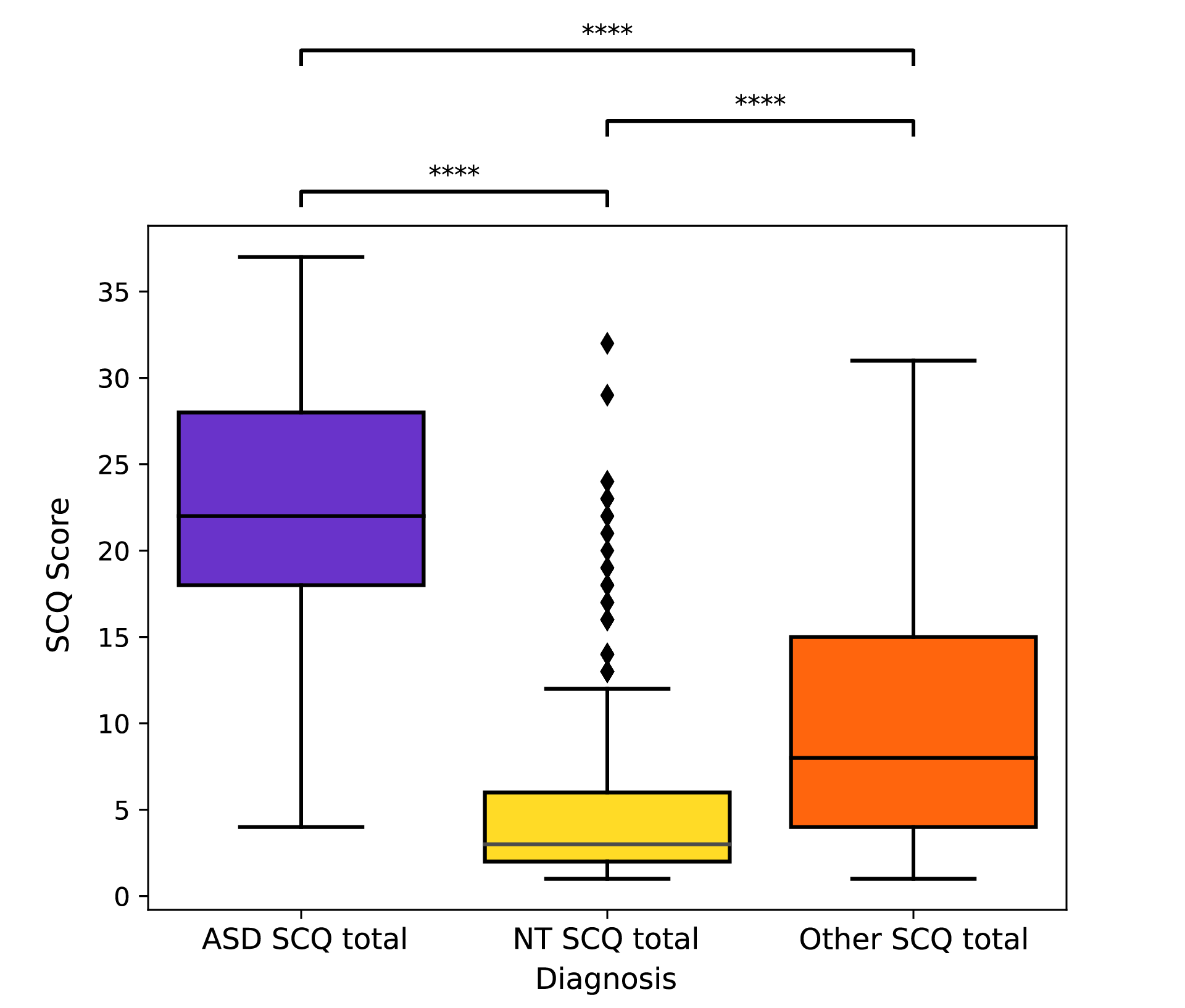


*Supplementary Figure 1.* ***Boxplot and statistical significance of diagnosis groups’ scores on ASD behavioral instrument validate parent-reported diagnosis.*** *We used two-way Mann-Whitney U tests to compare mean SCQ scores between children with ASD diagnoses and no diagnoses (p = 8.738e-98), children with no diagnoses and other diagnoses (p = 5.733e-23), and children with ASD and other diagnoses (p = 2.637e-66).*
